# Uptake of a national primary mental health program by young people in Australia

**DOI:** 10.1186/1752-4458-8-10

**Published:** 2014-03-21

**Authors:** Bridget Bassilios, Angela Nicholas, Lennart Reifels, Jane Pirkis

**Affiliations:** 1 Centre for Mental Health, Melbourne School of Population and Global Health, University of Melbourne, Victoria 3010, Australia

**Keywords:** Young people, Youth, Adolescents, Mental health services, Primary health care, Mental health policy

## Abstract

**Background:**

The purpose of this study was to examine the uptake of an Australian primary mental health care program (Access to Allied Psychological Services) by young people aged 12 to 25 years and the characteristics of consumers and the treatments received. Data were sourced from a national web-based minimum dataset.

**Results:**

Between 1 July 2003 and 30 June 2012, 51 716 young consumers received 245 704 sessions via the primary mental health program. Around two thirds were female and the average age was 19 years. The majority had depressive and/or anxiety disorders.

Most services were delivered to individuals (including just the young person and/or the young person with one or both parents), in a face-to-face context and free of charge. Cognitive and behavioral strategies were the most common interventions delivered.

**Conclusions:**

The primary mental health care program has been well utilized by people aged 12 to 25 years. Similar programs in other developed countries may improve access to primary mental health care for young people.

## Background

Youth mental health is an important public health problem in Australia and internationally. Approximately one in four young people have mental health problems but tend not to access services [1]. Primary mental health services have an important role to play in recognizing vulnerable individuals and offering appropriate treatment.

The Australian Government-funded Access to Allied Psychological Services (ATAPS) program is an example of a primary mental health care program that may improve access to mental health services for young people. Operating since 2001, ATAPS enables predominantly general practitioners (GPs; the Australian term for family physicians) to refer patients with high prevalence disorders (e.g., depression and anxiety) to mental health professionals (predominantly psychologists) for free or low-cost, evidence-based mental health care (most commonly cognitive behavioral therapy, or CBT). This care is delivered in up to 12 (or 18 in exceptional circumstances) individual and/or up to 12 group sessions. Review by the referring GP is essential after each block of six sessions and/or the final session [2]. Nation-wide, ATAPS is currently implemented by 61 Medicare Locals, which are regionally based organizations that coordinate primary health care delivery whilst striving to address local health care needs and service gaps.

The introduction of the Better Access to Psychiatrists, Psychologists and General Practitioners (Better Access) initiative in 2006 has influenced the nature and direction of ATAPS. Better Access facilitates similar access to primary mental health care via fee-for-service rebates under Medicare, Australia’s publicly funded universal health care system, operated by the government authority Medicare Australia [3]; however, unlike ATAPS its funding is uncapped. Consequently, ATAPS has since offered more flexible services to particular at-risk populations (e.g., people at risk of suicide, people affected by extreme climatic events, children with mental disorders) that are not available via either the original form of ATAPS, which operates simultaneously, or via Better Access.

ATAPS has been independently evaluated since its introduction, with findings indicating high program uptake in both urban and rural areas [4,5] and positive outcomes for consumers [6] and providers [7]. To date, however, the ATAPS evaluation has not specifically explored ATAPS’ performance in terms of providing care for young people as a particular at-risk population. The current paper, therefore, aims to describe the uptake of ATAPS by young people aged 12 to 25 years, the characteristics of this group of consumers and the interventions delivered to them. This age range was selected in order to be more inclusive given the variations in the definitions ‘young people’ by different services and researchers internationally, and to facilitate comparisons with other Australian youth mental health services, such as *headspace* National Youth Mental Health Foundation [8], which targets 12 to 25 year olds.

## Method

The ongoing evaluation of ATAPS has received approval from The University of Melbourne’s Human Research Ethics Committee.

### Data source

#### Minimum dataset

As per contractual requirements with the Australian Department of Health, data were collected by ATAPS service providers and entered by project officers (or providers) into a web-based purpose-designed national minimum dataset. The data includes the numbers of professionals and consumers involved in the program; socio-demographic (e.g., age, gender, level of income) and clinical (e.g., diagnosis, previous psychiatric service use) characteristics of consumers; the number, type and duration of sessions and the nature of the interventions provided. Data were downloaded from the minimum dataset on 29 October 2012.

### Data analyses

Consumers were included in the analyses if they were aged between 12 and 25 years at the time of referral. Descriptive analyses of the uptake of ATAPS, and consumer and session profiles were conducted using SPSS v21. The analysis period was 1 July 2003 to 30 June 2012.

## Results

### Uptake of ATAPS by young people

Between 1 July 2003 and 30 June 2012, a total of 66 264 referrals aged 12 to 25 years were made to ATAPS by 15 045 referrers (99% GPs), and sessions were conducted by 4 461 mental health professionals. This represents approximately 22% and 20% of all ATAPS referrals and sessions, respectively, during the analysis period. Overall, 245 704 sessions were reported in association with 51 716 (or 78% of the 66 264 referred) consumers who took up the service, making 4.8 the average number of sessions per referral.

**Figure 1:**
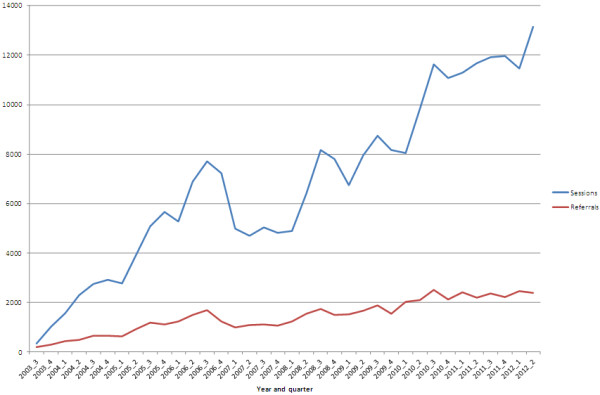
Referrals and sessions of care for young people over the life of ATAPS by quarter, July 2003 to June 2012.

Figure 1 shows the number of referrals (resulting in sessions) and the number of sessions delivered by quarter from July 2003 to June 2012. The number of referrals resulting in sessions and the number of sessions gradually increased and peaked in the third quarter of 2006 (1 681 and 7 699, respectively), after which there was a noticeable decline then a one-year period of stability before an overall ongoing pattern of increase. The noted decline is probably attributable to the introduction of the Better Access program in November 2006.

### Socio-demographic and clinical profile of consumers

Table 1 shows the socio-demographic and clinical characteristics of the 51 716 young people referred to ATAPS who received sessions. Over two-thirds of consumers were female, with an average age of 19 years. Approximately two-thirds of the young people (or their families) were receiving a low income, as determined by their GPs. Only one-third of consumers had previously accessed mental health care. Four percent of young consumers were reported to be of Aboriginal, and less than 1% of Torres Strait Islander, origin. The majority of consumers were diagnosed with depression (55%) and/or anxiety disorders (43%). ‘Other’ free text diagnoses were reported for 19% of consumers.

**Table 1:** Characteristics of young consumers who received care through ATAPS, July 2003 to June 2012 (N = 51 716)

	**Frequency**	**Percent**
**Gender**		
Female	35 590	68.8
Male	15 281	29.5
Missing	845	1.6
**Age (years)**		
12–14	5 929	11.5
15–17	10 819	20.9
18–21	17 411	33.7
22–25	17 557	33.9
**Low income**		
Yes	33 498	64.8
No	7 333	14.2
Unknown	7 794	15.1
Missing	3 091	6.0
**Previous psychiatric care**		
Yes	17 145	33.2
No	22 970	44.4
Unknown	7 543	14.6
Missing	4 058	7.8
**Aboriginal**		
Yes	2 055	4.0
No	40 635	78.1
Unknown	5 417	10.5
Missing	3 879	7.5
**Torres Strait Islander**^**a**^		
Yes	224	0.4
No	41 245	79.8
Unknown	5 693	11.0
Missing	4 554	8.8
**Diagnosis**^**b**^		
Alcohol and drug use disorders	2 480	4.8
Psychotic disorders	818	1.6
Depression	28 301	54.7
Anxiety disorders	22 128	42.8
Unexplained somatic disorders	870	1.7
Other	9 912	19.2
No formal diagnosis	50	0.1
Unknown	1 103	2.1
Missing	7 880	15.2

^a^Refers to the Indigenous people of the Torres Strait Islands, part of Queensland, Australia, as distinct from other Aboriginal peoples of Australia.

^b^Multiple responses permitted.

### Characteristics of sessions

The profile of sessions delivered to young consumers is shown in Table 2. Almost all sessions (92%) were delivered to individuals (including just the young person and/or the young person with one or both parents) in a face to face context (93%). Sessions of 46 to 60 minutes duration accounted for the vast majority (78%) of services delivered. The most frequently delivered interventions were CBT-cognitive (45%) and CBT-behavioral (35%); however, psycho-education (26%) and interpersonal therapy (21%) were also commonly delivered. Of the 179 852 sessions where payment information was available, the majority (142 840, 80%) were delivered free of charge. Around 9% of sessions were not attended.

**Table 2:** Summary characteristics of sessions provided to young consumers of ATAPS, July 2003 to June 2012 (N = 245 704)

	**Frequency**	**Percent**
**Duration**		
0–30 mins	7 276	3.0
31–45 mins	6 160	2.5
46–60 mins	192 452	78.3
Over 60 mins	22 745	9.3
Missing	17 071	6.9
**Type**		
Individual	222 592	90.6
Child	3 066	1.2
Parent(s)	94	0.0
Child & parent(s)	662	0.3
Group	5 620	2.3
Child in group	111	0.0
Parents in group	3	0.0
Missing	13 556	5.5
**Modality**		
Face to face	228 279	92.9
Telephone	1 678	0.0
Videoconference	131	0.1
Web-based	55	0.0
Missing	15 561	6.3
**Consumer fee**		
Yes	37 012	15.1
No	142 840	58.1
Missing	65 852	26.8
**Interventions**^**a**^		
Diagnostic assessment	41 834	17.0
Psycho-education	62 893	25.6
CBT Behavioural interventions	85 247	34.7
CBT Cognitive interventions	110 038	44.8
CBT Relaxation strategies	41 468	16.9
CBT Skills training	45 706	18.6
Interpersonal therapy	52 235	21.3
Narrative therapy	1 949	0.8
Family therapy	172	0.1
Parent training in behaviour management	77	0.0
Play therapy	82	0.0
Other	12 625	5.1
Missing	41 754	17.0

^a^Multiple responses permitted.

## Discussion

Substantially, 51 716 people aged 12 to 25 years received mental health care through ATAPS during the nine year analysis period. By comparison, and in the context that ATAPS funding is capped while Better Access funding is not, around 194 901 people aged 15–24 years received psychological therapy and focused psychological strategies in 2007–2009 via the Better Access program [9]. A further 14 548 consumers aged 12 to 25 years were referred to ATAPS but did not take up the service (22% of all referrals), which is similar to the non-uptake rate for the entire ATAPS program, at 21% [10], but lower than that (27%) reported elsewhere [11]. Previous findings have demonstrated that the likelihood of using services increases with symptom severity; [12] therefore it is possible that people referred to ATAPS but who do not take up the service have less severe symptoms. Other possible reasons for not taking up the service which could be further examined are lack of motivation or readiness to change; stigma associated with seeking mental health care; miscommunication with the GP; or access issues, such as the location or cost of the service.

The estimated Australian resident population aged 12 to 25 years in June 2012 was 4 081 058 [13], one quarter of whom had a potential need for mental health services [1]; given that 9 391 consumers in this age group accessed ATAPS in the 2011–2012 financial year, this equates to around 1% of consumers aged 12 to 25 years in potential need of mental health services accessing ATAPS. This rate needs to be interpreted in the context that ATAPS funding is capped and ATAPS is part of a suite of mental health services available for young people, such as Better Access, *headspace*, Orygen Youth Health and Child and Adolescent Mental Health Services. From 1 July 2003 to 30 June 2012, sessions delivered to people aged 12 to 25 years accounted for 20% of all ATAPS services. Given that people aged 12 to 25 years comprise around 18% of the Australian population [13], and that the prevalence of mental disorders is higher in younger age groups, the relative access to ATAPS by young consumers seems reasonable.

The fact that more females have taken up primary health care services via the ATAPS program is not surprising in the context that females with mental disorders in any group are more likely to use services than males [12]. This in turn may be attributable to multiple factors such as lower levels of stoicism and personal stigma associated with mental health problems in females compared with males [14], masculine norms being negatively associated with men’s willingness to seek professional help [15], and the advanced mental health literacy of young female Australians [16].

Most consumers were receiving low incomes or were from low income families and had not previously accessed psychiatric services, suggesting that the ATAPS program is addressing access barriers amongst those in need. Given that Indigenous people comprise 2.5% of the Australian population [17], the finding that around 4% of consumers were of Aboriginal, and less than 1% of Torres Strait Islander, origin indicates that they are appropriately accessing ATAPS. However, in the context that Indigenous people have a higher prevalence of mental disorders [18], their access to ATAPS could be improved. Indeed in July 2010, an ATAPS sub-program targeting Indigenous people was introduced that aims to address this inequity.

Findings based on the minimum dataset are limited by lack of corroboration of data such as diagnosis or intervention and may contain unidentified errors. For example, the ‘no show’ field was not mandatory until June 2013 and has a high rate of missing data, and so it is likely that this figure is currently underestimated and will increase in the future. However, there are several ways in which the quality of data in the minimum dataset is optimized including restriction of data entry options, mandatory data items, validation of data via code to ensure type of data entered matches type of data required and validation against data self-reported by Medicare Locals to the Department of Health, technical features to enable monitoring of each Medicare Local’s data patterns, data analysis checks, introduction of statistical linkage keys [19] to minimize duplicate patient entry, and provision of a helpdesk and website to support data entry.

Our findings are policy-relevant for both Australia and other developed countries with a similar primary mental health care system that are considering advancing that system. Our study corroborates previous findings suggesting that depressive and anxiety disorders are the most common mental disorders in young people [20]. The ATAPS model, which is predominantly reliant on a GP referral, is appropriate on the grounds that of the 35% of Australians with mental health problems who seek help, the majority do so from GPs [21]. Although the ATAPS program may not suit all young consumers given that access to services is reliant on their having a GP, ATAPS is a good example of a primary mental health care program which has been delivering services to a substantial number of young people with clinically diagnosable disorders and that facilitates access to free or low-cost treatment. Future evaluation efforts could examine spatial data to determine area-based equity in access to ATAPS by young people.

## Abbreviations

ATAPS: Access to Allied Psychological Services.

## Competing interests

During the study period, the evaluation of ATAPS was funded by the Australian Government’s Department of Health.

## Authors’ contributions

BB was the lead researcher for the study. She conducted the data analysis and interpretation and drafted the manuscript. AN and LR assisted with data interpretation and drafting the manuscript. JP oversaw the project and assisted with drafting the manuscript. All authors read and approved the final manuscript.
